# Genome sequence of the plant-growth-promoting bacterium Bacillus velezensis EU07

**DOI:** 10.1099/acmi.0.000762.v3

**Published:** 2024-05-30

**Authors:** Ömür Baysal, David J. Studholme, Catherine Jimenez-Quiros, Mahmut Tör

**Affiliations:** 1 Department of Molecular Biology and Genetics, Faculty of Science, Mugla Sitki Kocman University, 48000 Menteşe, Turkey; 2 Department of Biological Sciences, University of Worcester, Worcester, UK; 3 Biosciences, University of Exeter, Exeter, UK

**Keywords:** *Bacillus velezensis*, biological control, genome sequence, plant-growth promoting

## Abstract

Many Gram-positive spore-forming rhizobacteria of the genus *Bacillus* show potential as biocontrol biopesticides that promise improved sustainability and ecological safety in agriculture. Here, we present a draft-quality genome sequence for *Bacillus velezensis* EU07, which shows growth-promotion in tomato plants and biocontrol against *Fusarium* head blight. We found that the genome of EU07 is almost identical to that of the commercially used strain QST713, but identified 46 single-nucleotide differences that distinguish these strains from each other. The availability of this genome sequence will facilitate future efforts to unravel the genetic and molecular basis for EU07's beneficial properties.

## Data Summary

In this study, we generated genome sequence data, which has been deposited in public databases:

- National Center for Biotechnology Information (NCBI) BioProject accession number PRJNA743875 – https://www.ncbi.nlm.nih.gov/bioproject/743875
- Assembly NCBI GenBank accession number GCA_019997305.2 – https://www.ncbi.nlm.nih.gov/nuccore/JAIFZJ000000000
- NCBI RefSeq accession number GCF_019997305.2
- NCBI Sequence Read Archive (SRA) accession number SRR27184279.

## Introduction

Many Gram-positive spore-forming rhizobacteria of the genus *Bacillus* show potential as biocontrol biopesticides that promise improved sustainability and ecological safety in agriculture [1,3]. Here, we present genomic sequencing data for *Bacillus* strain Egem-Utku 07, hereafter known as EU07. This strain was previously isolated from the rhizosphere of diseased tomato plants [4] in an effort to collect strains that could inhibit the soilborne pathogen *Fusarium oxysporum* f. sp. *radicis-lycopersici* [4], which causes crown rot in tomato. We demonstrated that EU07 inhibits this pathogen *in vitro* [4]. Furthermore, EU07 promotes growth and inhibits fusarium head blight *in planta* [5]. We previously established that EU07 is a member of the genus *Bacillus*, but its precise species identity was ambiguous. Furthermore, in the absence of sequence data, little was known about the potential molecular mechanisms for its beneficial properties. Here, we present a draft-quality genome sequence assembly and genomic sequence reads from strain EU07. This dataset will help in better understanding EU07’s phylogeny and taxonomy, and provide a resource to assist elucidation of the molecular mechanisms of EU07’s beneficial traits.

## Methods

### Bacterial strain and isolation of genomic DNA

We isolated genomic DNA from bacterial strain EU07 from fresh liquid culture grown for 24 h in nutrient broth pH 7.2. We note that this medium provides a laboratory environment quite different from the bacterium’s normal soil environment. The liquid culture was inoculated from a single colony and, therefore, was assumed to be clonal. We used the ISOLATE II genomic DNA kit (Bioline), following the manufacturer’s instructions. The quality and concentration of the genomic DNA were assessed using a NanoDrop 2000c spectrophotometer (ThermoFisher Scientific).

### DNA sequencing

Genomic DNA was sent to the University of Exeter’s Sequencing Facility (https://biosciences.exeter.ac.uk/sequencing/) for Illumina Nextera XT library preparation and sequencing on the Illumina MiSeq platform to generate 748 528 pairs of 300 bp reads with a mean insert size of approximately 400 bp.

### Genome sequence assembly

We performed adapter trimming and quality filtering on the MiSeq reads using Trim Galore version 0.6.7 [6], which incorporates Cutadapt version 3.5 [7]. The -q parameter was set to 30 and we used the --paired option. The resulting cleaned read-pairs served as input for *de novo* assembly using SPAdes version 3.13.1 [8] with the --careful option. The resulting scaffolds and contigs were re-ordered against the reference genome of strain FZB42 with the Mauve Contig Mover [9]. Annotation was added by the National Center for Biotechnology Information (NCBI) Prokaryotic Genome Annotation Pipeline version 6.6 [10] after submission of the genome assembly. The command lines are documented in GitHub at https://github.com/davidjstudholme/bacillus_EU07/tree/main/assembly and in the Zenodo repository (https://doi.org/10.5281/zenodo.10968102) [11].

### Assessment of genome-assembly quality

We calculated assembly statistics using quast version 5.2.0 [12]. We checked read coverage of the genome assembly by aligning the EU07 reads against the EU07 assembly and calculating alignment statistics with Qualimap version 2.3 [13]. The alignment was performed using bwa-mem version 0.7.17 [14]; then, we reformatted and sorted the output using SAMtools version 1.13 [15]. The full details of the command lines are documented at https://github.com/davidjstudholme/bacillus_EU07/blob/main/assemblyQC/README.md and in the Zenodo repository [11].

### Average nucleotide identity (ANI)

We used fastANI [16] to calculate ANI between the genome of EU07 and each of the *Bacillus amyloliquefaciens* group (taxonomy ID: 1938374) genome assemblies retrieved from GenBank [17,18]. The exact command lines are documented in GitHub at https://github.com/davidjstudholme/bacillus_EU07/ and in the Zenodo repository [11].

### Phylogenomics

To generate a maximum-likelihood phylogenetic tree based on genome-wide SNPs, we used PhaME [19] with FastTree [20]. The exact command lines used are documented at https://github.com/davidjstudholme/bacillus_EU07/ and in the Zenodo repository [11]. The resulting tree was rendered using the Interactive Tree of Life (iTOL) 6.8.1 [21].

### Whole-genome alignment

Genome sequences were aligned using progressiveMauve version 2.4.0 [22] after first re-ordering the contigs against the reference genome of strain KNU-28 [23] with the Mauve Contig Mover [9]. The resulting alignment was visualized using Mauve snapshot_2015-02-25 [24]. The exact command lines used are documented at https://github.com/davidjstudholme/bacillus_EU07/ and in the Zenodo repository [11].

### Further whole-genome analyses

We used the Proksee web server [25] to perform several analyses of the assembled EU07 genome. This included blastn searches against 888 related genomes, annotation of horizontally acquired genomic regions with Alien Hunter [26], and identification of bacteriophage sequences using VirSorter [27,28] and Phigaro [29]. Variant-calling was performed using the Parsnp tool in Harvest [30].

## Results and discussion

### Genome sequencing and assembly

We generated 748 528 pairs of 300 bp Illumina MiSeq sequencing reads from EU07 genomic DNA. This represents approximately 100× coverage of the 4.2 Mbp genome. Trimming and filtering with Trim Galore left 715 442 pairs of reads, with lengths ranging from 20 to 300 bp. *De novo* assembly with SPAdes yielded 266 contigs with a total length of 4.2 Mbp and N_50_ length of 52.8 kb. This was deposited in GenBank via the NCBI Submission Portal under accession number GCA_019997305.2. The NCBI’s contamination filtering removed 5 contigs, leaving 261. The NCBI PGAP annotation system predicted 4 273 genes, of which 4 081 encode putative proteins. The results of NCBI’s quality check with CheckM v1.2.2 [31,32] revealed a completeness of 98.16 % (85th percentile) and 0.47 % contamination.

Alignment of sequencing reads against the genome assembly and analysis with Qualimap revealed a mean coverage of 93.25× and standard deviation of 89.87. Almost all of the genome assembly (99.96 %) had at least 1× coverage, and 97.59 % of the assembly had at least 10× coverage. The full Qualimap report and output files are available in the Zenodo repository (https://doi.org/10.5281/zenodo.10968102) [11], allowing users of this data to take coverage into account when performing analyses. We note that the contig with least coverage is JAIFZJ020000237.1, having only 1.04× coverage. Nevertheless, blast searches reveal that this contig shows very high levels of sequence similarity to genomes of other *Bacillus velezensis* strains, increasing confidence in its validity.

### EU07 belongs to the species *B. velezensis*

Previously, the phylogenetic and taxonomic position of strain EU07 had been ambiguous and we previously referred to it as ‘*B*. sp.’ and ‘*B. subtilis*’ [4,5]. To identify the species to which strain EU07 belongs, we uploaded the genome assembly to the Type Strain Genome Server (TYGS) [33]. This classified EU07 to the species *B. amyloliquefaciens*. Among the sequenced type strains in TYGS, the most similar to EU07 was FZB42 [34], which is the type strain of *B. amyloliquefaciens* subsp. *plantarum* [35]. However, this taxon is now considered to be synonymous with *B. velezensis* and distinct from *B. amyloliquefaciens* [36]. Hereafter, we refer to our strain as *B. velezensis* EU07.

### EU07 belongs to a clade of plant-associated strains of *B. velezensis*

**Fig. 1.**
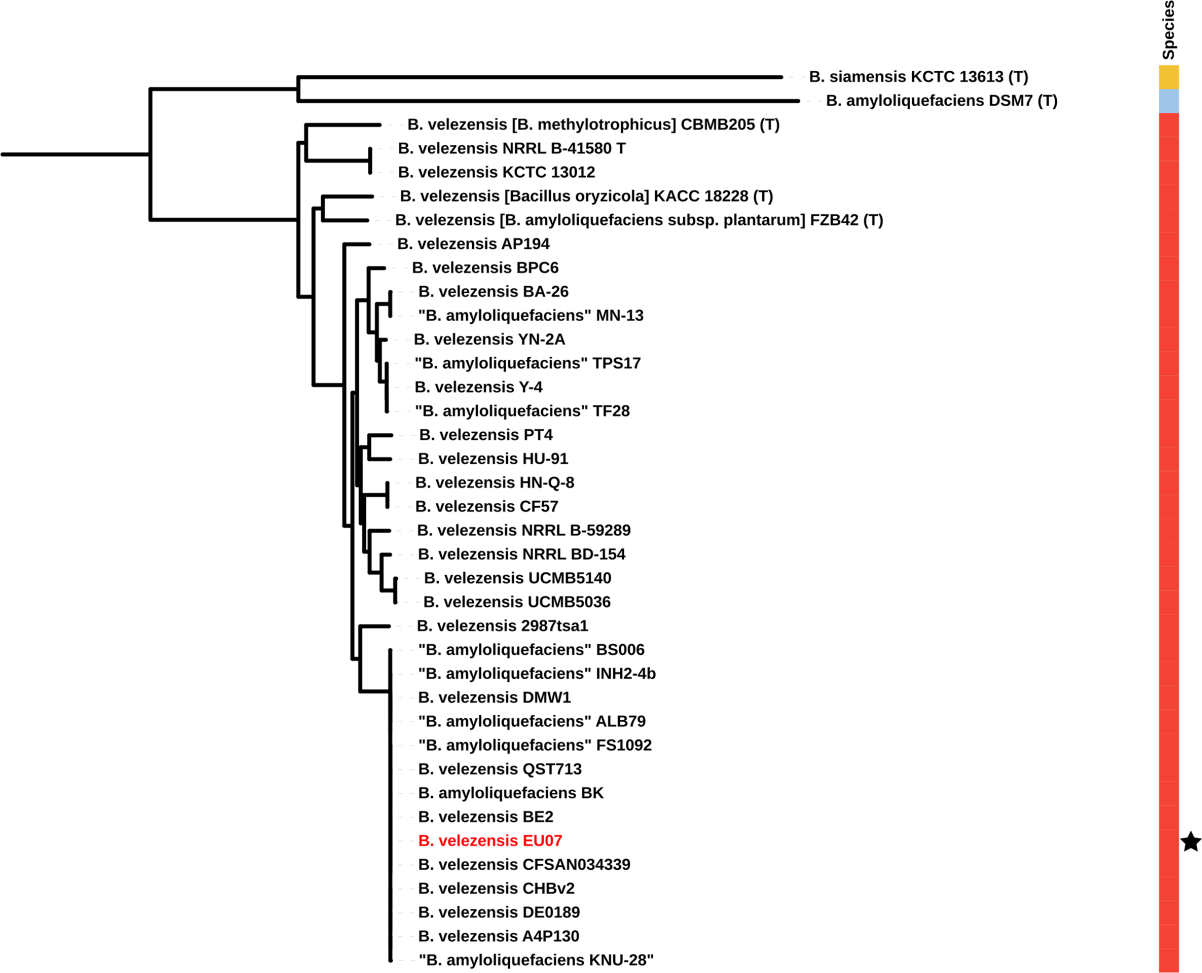
Phylogenetic position of *B. velezensis* EU07 within the *B. amyloliquefaciens* group. The phylogenomic maximum-likelihood tree was generated using PhaME and FastTree. The black star highlights the position of strain EU07, whose genome sequence is presented in this study. The configuration file and the tree files are deposited in GitHub at https://github.com/davidjstudholme/bacillus_EU07. Accession numbers for the genome assemblies can be found in Tables[Table T3] 3Tabl[Table T3]e 2Table 3. The tree can be viewed interactively at https://itol.embl.de/tree/14417323152242691702474608.

**Fig. 2.**
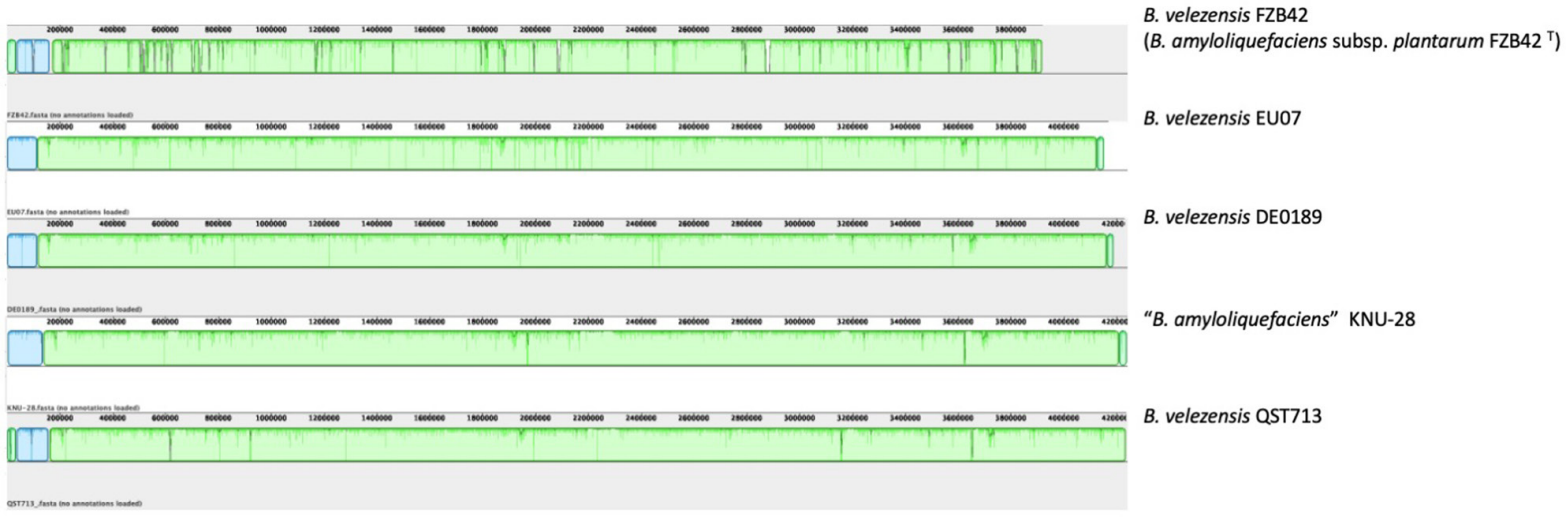
Whole-genome-sequence alignment between *B. velezensis* EU07 and closely related strains. Genome sequences were re-ordered, aligned and visualized using Mauve. Accession numbers for the genome assemblies can be found in Table 3. Green blocks in each genome are homologous to green blocks in all the other genomes. Blue blocks are homologous to the blue blocks in all the other genomes.

**Fig. 3.**
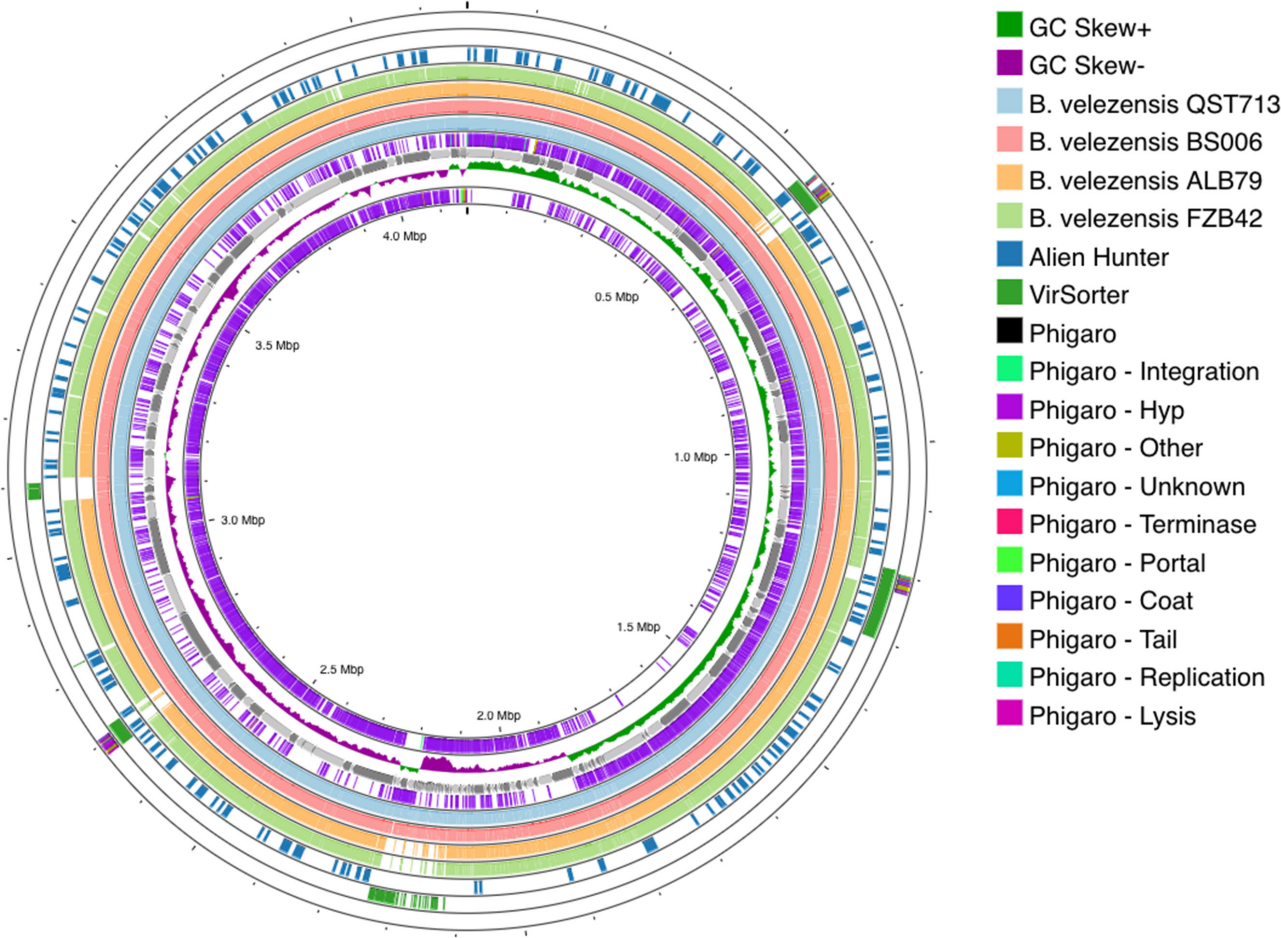
Overview of the genome of *B. velezensis* EU07 and comparison with closely related genomes. The circular plot of the EU07 chromosome was generated using Proksee. Data are arranged in nine concentric circular tracks as follows: (1) G+C skew, (2) EU07 contigs, (3) blastn hits against the QST713 genome, (4) blastn hits against the BS006 genome, (5) blastn hits against the ALB79 genome, (6) blastn hits against the FZB542 genome, (7) predicted horizontally acquired regions predicted by Alien Hunter, (8) phage loci predicted by VirSorter and (9) phage loci predicted by Phigaro.

To identify previously sequenced similar genomes, we calculated ANI between *B. velezensis* EU07 and all 888 genome assemblies available in GenBank for the *B. amyloliquefaciens* group. This revealed that EU07 shares more than 99.9 % ANI with 13 previously sequenced genomes. Table 1 lists the genomes showing the highest levels of ANI to that of *B. velezensis* EU07. This includes strains that previously have been classified variously as *B. amyloliquefaciens* or *B. velezensis*. However, they all fall within the *B. velezensis* clade [36,38] and should be considered as belonging to that species. To further elucidate the evolutionary relationships of EU07, we generated a phylogenomic tree including these closely related strains and the relevant type strains; this is presented in Fig. 1. Consistent with the ANI results, strain EU07 falls within a clade that includes the same 13 strains that showed greatest ANI with EU07. Alignment of these genomes with Mauve (Fig. 2) reveals extensive conservation and co-linearity of the chromosome sequence among these strains. Comparison of the EU07 chromosome versus the genome sequences of related strains, as shown in Fig. 3, revealed that most of the presence–absence polymorphism was associated with loci predicted to originate from bacteriophage genomes.

**Table 1.** Genomes that share more than 99 % ANI with *B. velezensis* EU07

GenBank accession no.	Reference	Strain	ANI (%)
GCA_004421045.1	[47]	**‘*B. amyloliquefaciens*’ FS1092**	99.99
GCA_021228895.1	[48]	***B. velezensis* A4P130**	99.99
GCA_003986895.1	–	***B. velezensis* BE2**	99.99
GCA_007678125.1	[49]	***B. velezensis* DE0189**	99.99
GCA_003073255.1	[37]	***B. velezensis* QST713**	99.99
GCA_026156445.1	[50]	***B. velezensis* CHBv2**	99.98
GCA_001709055.1	–	***B. velezensis* CFSAN034339**	99.98
GCA_019093835.1	–	**‘*B. amyloliquefaciens*’ BK**	99.98
GCA_014791945.1	–	**‘*B. amyloliquefaciens*’ INH2-4b**	99.98
GCA_028609625.1	[42]	***B. velezensis* DMW1**	99.98
GCA_003149795.1	[40]	**‘*B. amyloliquefaciens*’ ALB79**	99.95
GCA_024300805.1	[23]	**‘*B. amyloliquefaciens*’ KNU-28**	99.95
GCA_001278635.1	[39]	**‘*B. amyloliquefaciens*’ BS006**	99.94
GCA_024134605.1	–	*B. velezensis* 2987tsa1	99.12
GCA_000817575.1	[51]	‘*B. amyloliquefaciens*’ TF28	99.10
GCA_034060585.1	–	*B. velezensis* Y-4	99.07
GCA_010671715.1	[52]	*B. velezensis* HU-91	99.07
GCA_009193045.1	[53]	*B. velezensis* BPC6	99.07
GCA_034061945.1	–	*B. velezensis* YN-2A	99.05
GCA_026786545.1	–	*B*. velezensis NRRL B-59289	99.04
GCA_024138555.1	[54]	‘*B. amyloliquefaciens*’ TPS17	99.04
GCA_029866505.1	[55]	‘*B. amyloliquefaciens*’ MN-13	99.03
GCA_000341875.1	[56]	*B. velezensis* UCMB5036	99.02
GCA_009789615.1	[57]	*B. velezensis* BA-26	99.02
GCA_029910295.1	–	*B. velezensis* PT4	99.01
GCA_009738165.1	[58]	*B. velezensis* HN-Q-8	99.01
GCA_021559715.1	[59]	*B. velezensis* CF57	99.01
GCA_012647845.1	[60]	*B. velezensis* UCMB5140	99.01

Among the strains closely related to EU07 are several that previously have been described as having growth-promoting and/or pathogen-inhibitory properties. For example, strain BS006 was isolated from roots of *Physalis peruviana* in Colombia and promotes growth in banana [39]. Strain KNU-28 was isolated from peach leaves in Korea [23]. Strain ALB79 was isolated from grapes in northern California and shown to inhibit the growth of *Listeria monocytogenes in vitro* [40], while strain QST713 is used commercially (Serenade; Bayer) to protect mushroom crops against green mould disease and promotes growth in banana [37,41], among other applications. The endophytic *Bacillus* strain DMW1 was isolated from the inner tissues of potato tubers and exhibited strong biocontrol activity [42]. The near-identity of these genome sequences, independently isolated from plants in diverse geographical locations, suggests that EU07 is a member of a widely disseminated lineage of *B. velezensis* with biocontrol and growth-promoting properties. The molecular mechanisms and genetic determinants of these properties have been extensively reviewed elsewhere [43,45], and include gene clusters for secondary metabolites such as bacilysin, fengycin and macrolactin, which are conserved in the *B. velezensis* lineage that includes BS006 and EU07 [38].

Since our previous phenotypic comparisons between strains EU07 and QST713 revealed differences in their abilities to suppress fungal growth, we compared their genome sequences to identify possible genetic determinants of the observed differences. Their genomes are almost identical, with no detectable differences in their gene contents. However, we identified 46 single-nucleotide differences, which are listed in Table 2. These differences appear to be non-uniformly distributed across the genome. For example, 20 of the 46 SNPs occur within a single gene that encodes the beta subunit of a class-1b ribonucleoside-diphosphate reductase [46] (RefSeq WP_108702400.1; locus tag BVQ_RS09140). This suggests that these differences might be explained by recombination events associated with horizontal genetic transfer rather than point mutations. We also identified some sequence differences between EU07 and QST713 in the intergenic regions between several tRNA genes (GenBank accession no. JAIFZJ010000168.1). These genetic differences may explain the previously observed differences observed between the DNA fingerprints of these two strains when previously assayed using RAPDs [4].

**Table 2.** Forty-six SNPs between *B. velezensis* strains EU07 and QST713

Position in CP025079.1	Nucleotide in QST713	Nucleotide in EU07	Amino acid change	Predicted gene product
21 222	A	G	K→E	BVQ_RS00080: serine-tRNA ligase
230 096	A	C	E→A	BVQ_RS21890: non-ribosomal peptide synthetase
230 098	A	C	K→Q	BVQ_RS21890: non-ribosomal peptide synthetase
230 111	C	A	A→E	BVQ_RS21890: non-ribosomal peptide synthetase
530 737	T	G	Y→STOP	BVQ_RS02595: hypothetical protein
530 789	T	G	L→V	BVQ_RS02595: hypothetical protein
530 811	T	G	I→>S	BVQ_RS02595: hypothetical protein
531 288	T	G	I→S	BVQ_RS02595: hypothetical protein
705 298	A	C	F→V	BVQ_RS03655: GNAT family *N*-acetyltransferase
855 165	A	C	Non-coding	
1 168 486	A	C	Non-coding	
1 215 136	A	C	F→C	BVQ_RS06330: contact-dependent growth inhibition system immunity protein
1 851 920	T	G	F→L	BVQ_RS09140: class 1b ribonucleoside-diphosphate reductase subunit beta
1 851 923	A	T	G→G (synonymous)	BVQ_RS09140: class 1b ribonucleoside-diphosphate reductase subunit beta
1 851 925	C	A	T→K	BVQ_RS09140: class 1b ribonucleoside-diphosphate reductase subunit beta
1 851 929	G	T	K→N	BVQ_RS09140: class 1b ribonucleoside-diphosphate reductase subunit beta
1 851 932	A	G	E→E (synonymous)	BVQ_RS09140: class 1b ribonucleoside-diphosphate reductase subunit beta
1 851 935	A	G	Q→Q (synonymous)	BVQ_RS09140: class 1b ribonucleoside-diphosphate reductase subunit beta
1 851 938	C	T	D→D (synonymous)	BVQ_RS09140: class 1b ribonucleoside-diphosphate reductase subunit beta
1 851 941	T	G	T→T (synonymous)	BVQ_RS09140: class 1b ribonucleoside-diphosphate reductase subunit beta
1 851 944	T	C	Y→Y (synonymous)	BVQ_RS09140: class 1b ribonucleoside-diphosphate reductase subunit beta
1 851 950	A	G	K→K (synonymous)	BVQ_RS09140: class 1b ribonucleoside-diphosphate reductase subunit beta
1 851 953	T	G	V→V (synonymous)	BVQ_RS09140: class 1b ribonucleoside-diphosphate reductase subunit beta
1 851 954	T	C	L→L (synonymous)	BVQ_RS09140: class 1b ribonucleoside-diphosphate reductase subunit beta
1 851 956	A	C	L→F	BVQ_RS09140: class 1b ribonucleoside-diphosphate reductase subunit beta
1 851 959	T	C	A→A (synonymous)	BVQ_RS09140: class 1b ribonucleoside-diphosphate reductase subunit beta
1 851 962	A	C	G→G (synonymous)	BVQ_RS09140: class 1b ribonucleoside-diphosphate reductase subunit beta
1 851 965	T	G	L→L (synonymous)	BVQ_RS09140: class 1b ribonucleoside-diphosphate reductase subunit beta
1 851 969	T	C	L→L (synonymous)	BVQ_RS09140: class 1b ribonucleoside-diphosphate reductase subunit beta
1 851 971	A	G	L→L (synonymous)	BVQ_RS09140: class 1b ribonucleoside-diphosphate reductase subunit beta
1 851 972	T	C	L→L (synonymous)	BVQ_RS09140: class 1b ribonucleoside-diphosphate reductase subunit beta
1 851 974	G	T	L→F	BVQ_RS09140: class 1b ribonucleoside-diphosphate reductase subunit beta
1 878 004	T	G	Non-coding	
2 191 740	T	C	D→G	BVQ_RS10680: cysteine hydrolase family protein
2 415 378	C	A	Non-coding	
2 415 381	C	A	Non-coding	
2 415 440	C	A	Non-coding	
2 722 225	G	T	Non-coding	
2 722 243	T	G	Non-coding	
3 268 938	G	T	A→E	BVQ_RS16510: class 1 isoprenoid biosynthesis enzyme
3 269 022	T	G	N→T	BVQ_RS16510: class 1 isoprenoid biosynthesis enzyme
3 467 035	A	C	Non-coding	
3 489 562	A	G	F→F (synonymous)	BVQ_RS17685: lantibiotic immunity ABC transporter MutG family permease subunit
3 490 697	T	A	I→I (synonymous)	BVQ_RS17690: lantibiotic immunity ABC transporter MutE/EpiE family permease subunit
3 573 178	T	A	Non-coding	
4 000 822	T	G	Non-coding	

**Table 3. T3:** Genome sequences included in the phylogenomic analysis

GenBank accession no.	Taxon	Reference
GCA_003149795.1	‘*B. amyloliquefaciens*’ ALB79	[40]
GCA_019093835.1	‘*B. amyloliquefaciens*’ BK	–
GCA_001278635.1	‘*B. amyloliquefaciens*’ BS006	[39]
GCA_000196735.1	*B. amyloliquefaciens* DSM7^T^	[34]
GCA_004421045.1	‘*B. amyloliquefaciens*’ FS1092	[47]
GCA_014791945.1	‘*B. amyloliquefaciens*’ INH2-4b	–
GCA_024300805.1	‘*B. amyloliquefaciens*’ KNU-28	[23]
GCA_029866505.1	‘*B. amyloliquefaciens*’ MN-13	[55]
GCA_000817575.1	‘*B. amyloliquefaciens*’ TF28	[51]
GCA_024138555.1	‘*B. amyloliquefaciens*’ TPS17	[54]
GCA_000262045.1	*B. siamensis* KCTC 13613^T^	[61]
GCA_024134605.1	*B. velezensis* 2987tsa1	–
GCA_021228895.1	*B. velezensis* A4P130	[48]
GCA_001647965.1	*B. velezensis* AP194	[62]
GCA_009789615.1	*B. velezensis* BA-26	[57]
GCA_003986895.1	*B. velezensis* BE2	–
GCA_009193045.1	*B. velezensis* BPC6	[53]
GCA_003431885.1	*B. velezensis* (*B. methylotrophicus*) CBMB205^T^	[63]
GCA_021559715.1	*B. velezensis* CF57	[59]
GCA_001709055.1	*B. velezensis* CFSAN034339	–
GCA_026156445.1	*B. velezensis* CHBv2	[50]
GCA_007678125.1	*B. velezensis* DE0189	[49]
GCA_028609625.1	*B. velezensis* DMW1	[42]
GCA_000015785.2	*B. velezensis* (*B. amyloliquefaciens* subsp. *plantarum*) FZB42^T^	[34]
GCA_009738165.1	*B. velezensis* HN-Q-8	[58]
GCA_010671715.1	*B. velezensis* HU-91	[52]
GCA_001461835.1	*B. velezensis* (=*'B. oryzicola'*) KACC 18228^T^	[64]
GCA_001267695.1	*B. velezensis* KCTC 13012	[65]
GCA_001461825.1	*B. velezensis* NRRL B-41580^T^	[36]
GCA_026786545.1	*B. velezensis* NRRL B-59289	–
GCA_026787705.1	*B. velezensis* NRRL BD-154	–
GCA_029910295.1	*B. velezensis* PT4	–
GCA_003073255.1	*B. velezensis* QST713	[37]
GCA_000341875.1	*B. velezensis* UCMB5036	[56]
GCA_012647845.1	*B. velezensis* UCMB5140	[60]
GCA_034060585.1	*B. velezensis* Y-4	–
GCA_034061945.1	*B. velezensis* YN-2A	–
GCA_019997305.1	*B. velezensis* EU07	This study

### Conclusion

Genome sequencing of potential biocontrol strain EU07 revealed that it belongs to the species *B. velezensis*, a species often closely associated with plant roots, and well known for promoting plant growth and biocontrol. The EU07 strain is genetically almost identical to the commercially used strain QST713 (Serenade) and several other previously sequenced and characterized strains; however, we identified several genes containing single-nucleotide differences that can distinguish between EU07 and QST713. Strain EU07 is more distantly related to the commercially used *B. velezensis* strain FZB24 (TAEGRO), previously known as the type-strain of *B. amyloliquefaciens* subsp. *plantarum*. The availability of this genome sequence will facilitate future efforts to unravel the genetic and molecular basis for the strains beneficial properties.

## Abbreviations

ANI: average nucleotide identity
NCBI: National Center for Biotechnology Information

## References

[1] Saxena AK, Kumar M, Chakdar H, Anuroopa N, Bagyaraj DJ (2020). *Bacillus* species in soil as a natural resource for plant health and nutrition. J Appl Microbiol.

[2] Aloo BN, Makumba BA, Mbega ER (2019). The potential of bacilli rhizobacteria for sustainable crop production and environmental sustainability. Microbiol Res.

[3] Hashem A, Tabassum B, Fathi Abd Allah E (2019). *Bacillus subtilis*: a plant-growth promoting rhizobacterium that also impacts biotic stress. Saudi J Biol Sci.

[4] Baysal Ö, Çalışkan M, Yeşilova Ö (2008). An inhibitory effect of a new *Bacillus subtilis* strain (EU07) against *Fusarium oxysporum* f. sp. *radicis-lycopersici*. Physiol Mol Plant Pathol.

[5] Jimenez-Quiros C, Okechukwu EC, Hong Y, Baysal Ö, Tör M (2022). Comparison of antifungal activity of *Bacillus* strains against *Fusarium graminearum in vitro* and *in planta*. Plants.

[6] Krueger F http://www.bioinformatics.babraham.ac.uk/projects/trim_galore.

[7] Martin M (2011). Cutadapt removes adapter sequences from high-throughput sequencing reads. EMBnet J.

[8] Bankevich A, Nurk S, Antipov D, Gurevich AA, Dvorkin M (2012). SPAdes: a new genome assembly algorithm and its applications to single-cell sequencing. J Comput Biol.

[9] Rissman AI, Mau B, Biehl BS, Darling AE, Glasner JD (2009). Reordering contigs of draft genomes using the Mauve aligner. Bioinformatics.

[10] Tatusova T, DiCuccio M, Badretdin A, Chetvernin V, Nawrocki EP (2016). NCBI prokaryotic genome annotation pipeline. Nucleic Acids Res.

[11] Studholme DJ (2024). *Zenoob*.

[12] Gurevich A, Saveliev V, Vyahhi N, Tesler G (2013). QUAST: quality assessment tool for genome assemblies. Bioinformatics.

[13] Okonechnikov K, Conesa A, García-Alcalde F (2016). Qualimap 2: advanced multi-sample quality control for high-throughput sequencing data. Bioinformatics.

[14] Li H (2013). Aligning sequence reads, clone sequences and assembly contigs with BWA-MEM. arXiv.

[15] Li H, Handsaker B, Wysoker A, Fennell T, Ruan J (2009). The sequence alignment/map format and SAMtools. Bioinformatics.

[16] Jain C, Rodriguez-R LM, Phillippy AM, Konstantinidis KT, Aluru S (2018). High throughput ANI analysis of 90K prokaryotic genomes reveals clear species boundaries. Nat Commun.

[17] Benson DA (2004). GenBank. Nucleic Acids Research.

[18] Sayers EW, Cavanaugh M, Clark K, Ostell J, Pruitt KD (2019). GenBank. Nucleic Acids Res.

[19] Shakya M, Ahmed SA, Davenport KW, Flynn MC, Lo C-C (2020). Standardized phylogenetic and molecular evolutionary analysis applied to species across the microbial tree of life. Sci Rep.

[20] Price MN, Dehal PS, Arkin AP (2010). FastTree 2 – approximately maximum-likelihood trees for large alignments. PLoS One.

[21] Letunic I, Bork P (2021). Interactive Tree Of Life (iTOL) v5: an online tool for phylogenetic tree display and annotation. Nucleic Acids Res.

[22] Darling ACE, Mau B, Perna NT (2010). progressiveMauve: multiple genome alignment with gene gain, loss and rearrangement. PLoS One.

[23] Kim M-J, Park T, Jeong M, Lee G, Jung D-R (2022). Complete genome sequence of *Bacillus amyloliquefaciens* KNU-28 isolated from peach leaves (*Prunus persica* [L.] Batsch). Microbiol Resour Announc.

[24] Darling ACE, Mau B, Blattner FR, Perna NT (2004). Mauve: multiple alignment of conserved genomic sequence with rearrangements. Genome Res.

[25] Grant JR, Enns E, Marinier E, Mandal A, Herman EK (2023). Proksee: in-depth characterization and visualization of bacterial genomes. Nucleic Acids Res.

[26] Vernikos GS, Parkhill J (2006). Interpolated variable order motifs for identification of horizontally acquired DNA: revisiting the *Salmonella* pathogenicity islands. Bioinformatics.

[27] Roux S, Enault F, Hurwitz BL, Sullivan MB (2015). VirSorter: mining viral signal from microbial genomic data. PeerJ.

[28] Guo J, Bolduc B, Zayed AA, Varsani A, Dominguez-Huerta G (2021). VirSorter2: a multi-classifier, expert-guided approach to detect diverse DNA and RNA viruses. Microbiome.

[29] Starikova EV, Tikhonova PO, Prianichnikov NA, Rands CM, Zdobnov EM (2020). Phigaro: high-throughput prophage sequence annotation. Bioinformatics.

[30] Treangen TJ, Ondov BD, Koren S, Phillippy AM (2014). The Harvest suite for rapid core-genome alignment and visualization of thousands of intraspecific microbial genomes. Genome Biol.

[31] Parks DH, Imelfort M, Skennerton CT, Hugenholtz P, Tyson GW (2015). CheckM: assessing the quality of microbial genomes recovered from isolates, single cells, and metagenomes. Genome Res.

[32] Chklovski A, Parks DH, Woodcroft BJ, Tyson GW (2023). CheckM2: a rapid, scalable and accurate tool for assessing microbial genome quality using machine learning. Nat Methods.

[33] Meier-Kolthoff JP, Göker M (2019). TYGS is an automated high-throughput platform for state-of-the-art genome-based taxonomy. Nat Commun.

[34] Rückert C, Blom J, Chen X, Reva O, Borriss R (2011). Genome sequence of *B. amyloliquefaciens* type strain DSM7(T) reveals differences to plant-associated *B. amyloliquefaciens* FZB42. J Biotechnol.

[35] Borriss R, Chen X-H, Rueckert C, Blom J, Becker A (2011). Relationship of *Bacillus amyloliquefaciens* clades associated with strains DSM 7T and FZB42T: a proposal for *Bacillus amyloliquefaciens* subsp. *amyloliquefaciens* subsp. nov. and *Bacillus amyloliquefaciens* subsp. *plantarum* subsp. nov. based on complete genome sequence comparisons. Int J Syst Evol Microbiol.

[36] Dunlap CA, Kim S-J, Kwon S-W, Rooney AP (2016). *Bacillus velezensis* is not a later heterotypic synonym of *Bacillus amyloliquefaciens*; *Bacillus methylotrophicus*, *Bacillus amyloliquefaciens* subsp. *plantarum* and ‘*Bacillus oryzicola*’ are later heterotypic synonyms of *Bacillus velezensis* based on phylogenomics. Int J Syst Evol Microbiol.

[37] Pandin C, Le Coq D, Deschamps J, Védie R, Rousseau T (2018). Complete genome sequence of *Bacillus velezensis* QST713: a biocontrol agent that protects *Agaricus bisporus* crops against the green mould disease. J Biotechnol.

[38] Palazzini JM, Dunlap CA, Bowman MJ, Chulze SN (2016). *Bacillus velezensis* RC 218 as a biocontrol agent to reduce *Fusarium* head blight and deoxynivalenol accumulation: genome sequencing and secondary metabolite cluster profiles. Microbiol Res.

[39] Gamez RM, Rodríguez F, Bernal JF, Agarwala R, Landsman D (2015). Genome sequence of the banana plant growth-promoting rhizobacterium *Bacillus amyloliquefaciens* BS006. Genome Announc.

[40] Tran TD, Huynh S, Parker CT, Hnasko R, Gorski L (2018). Complete genome sequences of three *Bacillus amyloliquefaciens* strains that inhibit the growth of *Listeria monocytogenes in vitro*. Genome Announc.

[41] Tian L, Zhang W, Zhou G-D, Li S, Wang Y (2023). A biological product of *Bacillus amyloliquefaciens* QST713 strain for promoting banana plant growth and modifying rhizosphere soil microbial diversity and community composition. Front Microbiol.

[42] Yu C, Chen H, Zhu L, Song Y, Jiang Q (2023). Profiling of antimicrobial metabolites synthesized by the endophytic and genetically amenable biocontrol strain *Bacillus velezensis* DMW1. Microbiol Spectr.

[43] Chowdhury SP, Hartmann A, Gao X, Borriss R (2015). Biocontrol mechanism by root-associated *Bacillus amyloliquefaciens* FZB42 – a review. Front Microbiol.

[44] Chen XH, Koumoutsi A, Scholz R, Schneider K, Vater J (2009). Genome analysis of *Bacillus amyloliquefaciens* FZB42 reveals its potential for biocontrol of plant pathogens. J Biotechnol.

[45] Magno-Pérez-Bryan MC, Martínez-García PM, Hierrezuelo J, Rodríguez-Palenzuela P, Arrebola E (2015). Comparative genomics within the *Bacillus* genus reveal the singularities of two robust *Bacillus amyloliquefaciens* biocontrol strains. Mol Plant Microbe Interact.

[46] Zhang Y, Stubbe J (2011). *Bacillus subtilis* class Ib ribonucleotide reductase is a dimanganese(III)-tyrosyl radical enzyme. Biochemistry.

[47] Gu G, Gonzalez-Escalona N, Zheng J, Bolten S, Luo Y (2020). Genome sequences of *Brevundimonas naejangsanensis* strain FS1091 and *Bacillus amyloliquefaciens* strain FS1092, isolated from a fresh-cut-produce-processing plant. Microbiol Resour Announc.

[48] Becker R, Ulrich K, Behrendt U, Schneck V, Ulrich A (2022). Genomic characterization of *Aureimonas altamirensis* C2P003 – a specific member of the microbiome of *Fraxinus excelsior* trees tolerant to ash dieback. Plants.

[49] Zhang C, Song W, Ma HR, Peng X, Anderson DJ (2020). Temporal encoding of bacterial identity and traits in growth dynamics. Proc Natl Acad Sci USA.

[50] Nøhr-Meldgaard K, Struve C, Ingmer H, Agersø Y (2022). Intrinsic tet(L) sub-class in *Bacillus velezensis* and *Bacillus amyloliquefaciens* is associated with a reduced susceptibility toward tetracycline. Front Microbiol.

[51] Zhang S, Jiang W, Li J, Meng L, Cao X (2016). Whole genome shotgun sequence of *Bacillus amyloliquefaciens* TF28, a biocontrol entophytic bacterium. Stand Genomic Sci.

[52] Wash P, Yasmin H, Ullah H, Haider W, Khan N (2023). Deciphering the genetics of antagonism and antimicrobial resistance in *Bacillus velezensis* HU-91 by whole genome analysis. J King Saud Univ Sci.

[53] Sun W, Yan L, Chen C, Tian Y, Li X (2020). Identification and biocontrol effect of antagonistic bacterium *Bacillus velezensis* Bpc6 against soft rot and *Sclerotinia* rot diseases on lettuce. Chin J Biol.

[54] Kuebutornye FKA, Lu Y, Abarike ED, Wang Z, Li Y (2020). In vitro assessment of the probiotic characteristics of three *Bacillus* species from the gut of Nile Tilapia, *Oreochromis niloticus*. Probiotics Antimicrob Proteins.

[55] Yang J, Gao MY, Li M, Li ZZ, Li H (2018). *Bacillus amyloliquefaciens* CotA degradation of the lignin model compound guaiacylglycerol-β-guaiacyl ether. Lett Appl Microbiol.

[56] Manzoor S, Niazi A, Bejai S, Meijer J, Bongcam-Rudloff E (2013). Genome sequence of a plant-associated bacterium, *Bacillus amyloliquefaciens* strain UCMB5036. Genome Announc.

[57] Wang B, Liu C, Yang X, Wang Y, Zhang F (2021). Genomics-guided isolation and identification of active secondary metabolites of *Bacillus velezensis* BA-26. Biotechnol Biotechnol Equip.

[58] Zhao J, Zhou Z, Bai X, Zhang D, Zhang L (2022). A novel of new class II bacteriocin from *Bacillus velezensis* HN-Q-8 and its antibacterial activity on *Streptomyces scabies*. Front Microbiol.

[59] Zhang C, Chen L, Si H, Gao W, Liu P (2020). Study on the characteristics and mechanisms of nicosulfuron biodegradation by *Bacillus velezensis* CF57. J Basic Microbiol.

[60] Reva ON, Larisa SA, Mwakilili AD, Tibuhwa D, Lyantagaye S (2020). Complete genome sequence and epigenetic profile of *Bacillus velezensis* UCMB5140 used for plant and crop protection in comparison with other plant-associated *Bacillus* strains. Appl Microbiol Biotechnol.

[61] Jeong H, Jeong D-E, Kim SH, Song GC, Park S-Y (2012). Draft genome sequence of the plant growth-promoting bacterium *Bacillus siamensis* KCTC 13613T. J Bacteriol.

[62] Hassan MK (2021). In vitro pectate lyase activity and carbon uptake assays and whole genome sequencing of *Bacillus amyloliquefaciens* subsp. *plantarum* strains for a pectin defective pathway. bioRxiv.

[63] Hwangbo K, Um Y, Kim KY, Madhaiyan M, Sa TM (2016). Complete genome sequence of *Bacillus velezensis* CBMB205, a phosphate-solubilizing bacterium isolated from the rhizoplane of rice in the Republic of Korea. Genome Announc.

[64] Chung EJ, Hossain MT, Khan A, Kim KH, Jeon CO (2015). *Bacillus oryzicola* sp. nov., an endophytic bacterium isolated from the roots of rice with antimicrobial, plant growth promoting, and systemic resistance inducing activities in rice. Plant Pathol J.

[65] Jeong H, Park S-H, Choi S-K (2015). Genome sequence of antibiotic-producing *Bacillus amyloliquefaciens* strain KCTC 13012. Genome Announc.

